# How long can tardigrades survive in the anhydrobiotic state? A search for tardigrade anhydrobiosis patterns

**DOI:** 10.1371/journal.pone.0270386

**Published:** 2023-01-11

**Authors:** Milena Roszkowska, Bartłomiej Gołdyn, Daria Wojciechowska, Zofia Księżkiewicz, Edyta Fiałkowska, Mateusz Pluskota, Hanna Kmita, Łukasz Kaczmarek

**Affiliations:** 1 Faculty of Biology, Department of Animal Taxonomy and Ecology, Adam Mickiewicz University, Poznań, Poland; 2 Faculty of Biology, Department of Bioenergetics, Institute of Molecular Biology and Biotechnology, Adam Mickiewicz University, Poznań, Poland; 3 Faculty of Biology, Department of General Zoology, Adam Mickiewicz University, Poznań, Poland; 4 Faculty of Physics, Department of Biomedical Physics, Adam Mickiewicz University, Poznań, Poland; 5 Institute of Environmental Sciences, Jagiellonian University, Kraków, Poland; University of Colorado Boulder, UNITED STATES

## Abstract

Anhydrobiosis is a desiccation tolerance that denotes the ability to survive almost complete dehydration without sustaining damage. The knowledge on the survival capacity of various tardigrade species in anhydrobiosis is still very limited. Our research compares anhydrobiotic capacities of four tardigrade species from different genera, i.e. *Echiniscus testudo*, *Paramacrobiotus experimentalis*, *Pseudohexapodibius degenerans* and *Macrobiotus pseudohufelandi*, whose feeding behavior and occupied habitats are different. Additionally, in the case of *Ech*. *testudo*, we analyzed two populations: one urban and one from a natural habitat. The observed tardigrade species displayed clear differences in their anhydrobiotic capacity, which appear to be determined by the habitat rather than nutritional behavior of species sharing the same habitat type. The results also indicate that the longer the state of anhydrobiosis lasts, the more time the animals need to return to activity.

## Introduction

Cryptobiosis is a latent state of life in which no signs of an organism’s activity are visible [1]. It is an adaptation to harsh environmental conditions that allows organisms to survive periods unsuitable for active life, such as lack of water or very low temperatures. The phenomenon of cryptobiosis has been observed in unicellular, e.g. bacteria and protists, as well as multicellular organisms, including lichens, liverworts, vascular plants, as well as, certain invertebrate animals. Several different types of cryptobiosis have been described, all of which were induced by specific stress conditions, such as anhydrobiosis (lack of water), cryobiosis (low temperature), anoxybiosis (lack of oxygen) or osmobiosis (change of osmotic conditions) [e.g. 2–4]. Among them the most studied form of cryptobiosis is anhydrobiosis [e.g. 5–10].

Anhydrobiosis is defined as dehydration tolerance, which is the ability of a species to survive almost complete loss of body water. This adaptation strategy is important for mosses, lichens, liverworts in habitats characterized by irregular water availability, such as soil, temporary ponds or puddles, cryoconite holes or tree holes [e.g. 11–14]. In multicellular organisms, anhydrobiosis may occur at particular stages of development, but it is rarely observed in adulthood [e.g. 2–4, 6, 8, 15, 16]. Some organisms may stay in an anhydrobiotic state for a very long time [17] and later they may be rehydrated and return to active life. Entering anhydrobiosis is a high-cost strategy for an organism, demanding delivery of extra energy accumulated in specially adapted cells, called storage cells [18, 19]. According to previous studies, the longer an organism stays in the dehydrated state, the more time it requires to return to full activity, and surpassing the dehydration time threshold can be lethal [e.g. 20–23]. This increased mortality after extended periods of anhydrobiosis may result from an inefficient energy supply and/or insufficiency of relevant cellular protection/repair mechanisms [10, 16, 18, 19].

Water bears (Tardigrada), roundworms (Nematoda), and wheel animals (Rotifera) are known to be the most effective species at anhydrobiosis, regardless of their stage in life [e.g. 6–9, 24, 25]. In fact, this process has been studied best in tardigrades out of all phyla. In Tardigrada, anhydrobiosis is divided into three stages. The first stage involves dehydration. The second is known as the “tun” and it represents a fully desiccated animal. The third stage–rehydration–occurs when water becomes accessible for the tardigrade again. To date, experiments on anhydrobiosis in controlled laboratory conditions have been conducted for several species, including *Acutuncus antarcticus* (Richters, [26]) [27], *Bertolanius volubilis* (Durante Pasa & Maucci, [28]) [29], *Echiniscus testudo* (Doyère, [30]) [31], *Hypsibius exemplaris* Gąsiorek Stec, Morek & Michalczyk, [32] (formerly *Hys*. *dujardini* (Doyère, [30]) [33–42]), *Milnesium inceptum* Morek, Suzuki, Schill, Georgiev, Yankova, Marley & Michalczyk, [43] (formerly *Mil*. *tardigradum* (Doyère, [30]) [25, 31, 41, 44–46]), *Paramacrobiotus richtersi* (Murray, [47]) [48, 49], *Pam*. *spatialis* Guidetti, Cesari, Bertolani, Altiero & Rebecchi, [50, 51], *Ramazzottius oberhaeuseri* (Doyère, [30]) [52, 53], *Ram*. *varieornatus* (Bertolani & Kinchin, [54]) [34, 37, 55, 56] and *Richtersius coronifer* (Richters, [57]) [52, 53, 58–60]. However, the results of those experiments cannot be directly compared due to differences in the applied protocols of dehydration, duration of the tun and the time window of the revival. That is why distinct patterns of tardigrade anhydrobiosis cannot be distinguished.

Nevertheless, it is assumed that aquatic tardigrades are less efficient in anhydrobiosis than the limno-terrestrial ones [e.g. 2, 20, 61]. Since tardigrade tuns may be easily spread by wind or vertebrate species, e.g. birds [62, 63], the possibility of colonizing new areas exposed to periodic drying [64] may be a good explanation for this difference. On the other hand, differences in anhydrobiotic capabilities were also observed among species occupying the same habitat [65], but representing different classes, such as Heterotardigrada and Eutardigrada, or with different life strategies [22, 65]. This corresponds to an observation that optimal air humidity and temperature for dehydration and the duration of the tun stage not only may differ between different species [e.g. 2, 20], but also within the same species [e.g. 21, 33, 66].

Most of the published studies on the anhydrobiotic abilities of tardigrade species have focused on their ability to enter anhydrobiosis for a short time (from a few hours to 1–3 days). These studies primarily focused on testing the survival of tardigrades in environmental extremes, including radiation, changes in humidity, and high temperatures, or repeated anhydrobiosis at different life stages. The studies included research on DNA repair mechanisms and stress proteins synthesis on the basis of genomic analyses [21, 25, 27, 29, 33–35, 37–41, 45, 46, 48, 51, 55, 59, 60]. The conclusion from these studies shows that different tardigrade species have a high degree (80–90%) of survival after short periods of anhydrobiosis.

Experiments involving slightly longer duration of the tun stage (7 days) were conducted by Hengherr et al. [44] for *Milnesium inceptum* (former *Mil*. *tardigradum*). Those specimens were able to survive dozens of cycles of rehydration from the tun stage followed by dehydration for 7 days. When compared with a hydrated control group, the periodically dried animals showed a similar longevity as the control group. This study showed that the time spent in the tun stage is not “counted” by the animals and supports the “Sleeping Beauty” hypothesis [44]. A 7-day tun stage was also applied in experiments designed to test thermo-tolerance of anhydrobiotic *Ram*. *varieornatus* [56]. Control groups not exposed to high temperatures had a survival rate of 96–99%, however, their survivability declined drastically if the tuns were kept in temperatures higher than 55°C. Another 7-day tun stage experiment was conducted on *Hys*. *exemplaris*, showing differences in survivability between naturally and artificially dried specimens. However, the overall survivability of the studied specimens was rather low and oscillated between a few percent and 50% [42].

A 12-day tun stage was tested on Italian and Swedish populations of *Ram*. *oberhauseri* and *Ric*. *coronifer*, where similar survival rates were observed for the same species from different locations (approx. 40% for *Ric*. *coronifer* and approx. 66% for *Ram*. *oberhaeuseri*). Another study showed that body size strongly effected the possibility of returning from anhydrobiosis, but the effect had the opposite direction in the case of the studied species [52]. The latter study was confirmed by Faurby et al. [53], except some differences in survival rates between *Ram*. *oberhaeuseri* from Swedish and Italian populations, whereas no such differences were found for *Ric*. *coronifer*. The authors suggested that a geographic variation in successful anhydrobiosis may be a general feature. Furthermore, studies based on a 12-day tun stage showed that medium-sized tardigrades exhibiting better energetic condition enjoyed higher survival rates than larger specimens [58].

Experiments on the influence of hypomagnetic conditions on *Ech*. *testudo*, *Hys*. *exemplaris* and *Mil inceptum* undergoing anhydrobiosis showed a very high survival rate after 21 days in anhydrobiosis for *Ech*. *testudo* and *Mil inceptum* (>70%) and very low for *Hys*. *exemplaris* (20–26%) in control groups not exposed to hypmagnetic conditions. Specimens exposed to hypomagnetic conditions had survival rates of 54–68% for *Ech*. *testudo* and *Mil inceptum*, and 2–20% for *Hys*. *exemplaris* [36, 66]. Similarly low survival rates involved *Hys*. *exemplaris* in 7-day experiments performed by Poprawa et al. [42], who also suggested that it was probably a naturally occurring situation for that freshwater species. In contrast to *Hys*. *exemplaris*, a very high survival rate (~ 90%) was reported for *Mil*. *inceptum* after 30 and 60 days in anhydrobiosis [41].

Another category of studies focused on tardigrade anhydrobiosis featured experiments conducted in outer space. Anhydrobiotic *Ric*. *coronifer*, *Ram*. *oberhauseri* and *Ech*. *testudo* exposed to space vacuum for two weeks were unambiguously able to return to active life. However, after two years in the dehydrated state, none of the tardigrades exposed to cosmic radiation returned to life [67]. Similarly to laboratory experiments involving high doses of radiation and low temperatures where tardigrades were desiccated in moss substrate together with rotifers and nematodes, the survival rate was rather low [67]. Moreover, Rebecchi et al. [49] tested anhydrobiotic *Pam*. *richtersi* during TARSE (Tardigrade Resistance to Space Effects), measuring their survival rate after two weeks. They found that survival was very high and differed depending on whether animals were dehydrated in leaf litter (78.9%) or on a paper (94.4%). However, in those experiments the drying protocols for the two comparators were not identical and were not standardized for all the species, and the anhydrobiosis time was relatively short.

Anhydrobiosis of tardigrades that was much longer and successful was reported in several other papers. In a long-term, semi-natural experiment, tardigrades were desiccated in lichen samples and stored in ambient laboratory conditions. The lichen sample was checked 20 times over 1,604 days (4 years and 5 months), and the survival rate was calculated for eutardigrade *Ram*. *oberhaeuseri*, two heterotardigrade species *Ech*. *trisetosus* [68] and *Ech*. *testudo* [22]. During that time, *Ram*. *oberhaeuseri* and *Echiniscus* spp. experienced decreased survivability from 91% and 72% at the beginning of the experiment, respectively, to almost 0% at the end. Thus, specimens of *Ram*. *oberhaeuseri* survived up to 1,604 days, while *Echiniscus* spp. up to 1,085 days. Baumann [69] reported successful anhydrobiosis of *Macrobiotus* [70] species after almost 7 years in the tun stage. Much later, Guidetti and Jönsson [71] analysed eggs of *Ramazzottius* [72] from 9-year-old dried moss and lichen samples which hatched successfully and survived for up to 40 days. Then, Roszkowska et al. [17] reported successful survival of a tun stage lasting 12 and 15 years for *Mac*. cf. *hufelandi* and *Mil*. *argentinum* (Roszkowska, Ostrowska & Kaczmarek) [73], respectively. However, the longest and best-documented survival from the tun stage was reported for heterotardigrade *Ech*. *testudo* which was stored dehydrated for approx. 20 years [74]. Unfortunately, most of those experiments (except [22]) were anecdotal observations of single specimens which were not conducted in strict laboratory conditions. They involved stored dried moss or lichen containing tardigrades, and the experiments consisted merely of checking whether any individuals would return to activity after an extended period in the dry state. Therefore, it is not possible to infer from them any patterns of anhydrobiosis.

In order to obtain comparable results for different species, also those living in different habitats, we used the same standardized and controlled conditions. The only available relevant study conducted in such laboratory conditions was performed by Roszkowska et al. [65] for *Mil*. *inceptum* and *Ram*. *subanomalus* (Biserov, [75]), representing predatory and herbivorous species, respectively, that often co-occurred in the same habitat. The obtained results supported a conclusion that carnivorous species displayed better anhydrobiosis survivability than the herbivorous ones. Therefore, in the same habitat, predatory and prey species may follow different anhydrobiosis patterns. For this reason, we focused on anhydrobiosis patterns for tardigrade species that differed in feeding behavior, habitat preference, and taxonomic status using a long-term experiment (up to 240 days) in standardized laboratory conditions. The studied organisms were obtained from five tardigrade populations (four species) representing Heterotardigrada and Eutardigrada. To provide for differences between species inhabiting divergent habitats, three species from Poland and one from Madagascar were used. Additionally, one species was represented by specimens from two separate populations: an urban population, and a population collected from a national park approx. 12 km away from the urban one. Then, one of the species was cultured continuously in laboratory conditions, whereas the others were collected from environmental samples. This approach allowed for a comparison of anhydrobiotic capabilities of several tardigrade species in the same, standardised laboratory conditions so as to eliminate any interference from various uncontrolled experimental factors. The obtained results indicate that anhydrobiosis survival is very high or high for the tun stage up to 120 days, even though clear differences in the anhydrobiosis capabilities between different species/populations increase with the tun stage duration.

## Material and methods

### Species used in experiment and sample processing

Heterotardigrade *Ech*. *testudo* (populations A and B) and Eutardigrada *Paramacrobiotus experimentalis* Kaczmarek, Mioduchowska, Poprawa & Roszkowska, [76], *Pseudohexapodibius degenerans* (Biserov, [77]) and *Macrobiotus pseudohufelandi* Iharos, [78] were found in moss and soil samples (Table 1). All specimens used in experiments were extracted directly from environmental samples, except *Pam*. *experimentalis*, which had been cultured in laboratory conditions since 2019 (as described in [79]); those specimens were extracted from the stock culture.

**Table 1:** Species used in experiments and their feeding behaviour and localities.

Species	Feeding behaviour	Sample type	Locality	Coordinates	Altitude
*Ech*. *testudo* (A)	herbivorous	moss on railway embankment	Wielkopolski National Park, Poland	52°19’09"N; 16°48’23"E	86 m asl
*Ech*. *testudo* (B)	herbivorous	moss on concrete wall	Przybyszewskiego street, Poznań, Poland	52°24’15"N; 16°53’18"E	87 m asl
*Pam*. *experimentalis*	predatory	moss on soil	Toamasina Province, near Ambavaniasy, Madagascar	18°56′37″S; 48°30′52″E	717 m asl
*Psh*. *degenerans*	herbivorous	soil between grass roots	Słowiński National Park, Poland	54°44′54.91′′N; 17° 26′14.83′′E	8 m asl
*Mac*. *pseudohufelandi*	herbivorous	soil between grass roots	Morasko University Campus, Poznań, Poland	52°28′04.70′′N; 16° 55′45.47′′E	88 m asl

Moss samples were placed into plastic beakers containing 250 ml of tap water. After 18 hours, the water-saturated moss was strongly shaken with tweezers and all plant particles were removed. Water with tardigrades was then poured into a 250 ml plastic cylinder, and left to settle for 30 minutes, after which the upper portion of water (ca. 200 ml) was decanted and discarded and the remaining 50 ml was poured into Petri dishes for tardigrade extraction under a stereomicroscope (Olympus SZ). Soil samples with grass were placed into plastic beakers containing 1000 ml of tap water. After 6–8 hours, the water-saturated soil and grass were strongly shaken with tweezers and then water with floating organic particles was immediately poured through a limnological net with 40 μm mesh size (leaving the grains of sand at the bottom of the beaker). Organic material remaining on the mesh was rinsed with water into a 500 ml beaker and then poured into two 250 ml plastic cylinders. After 30 minutes, the upper portion of water (ca. 200 ml) from each cylinder was decanted and discarded and the remaining 50 ml was poured into Petri dishes for tardigrade extraction under a stereomicroscope (Olympus SZ).

Specimens were later sorted and only fully active, adult specimens of similar, moderate body length were selected for the anhydrobiosis experiment. Genus abbreviations follow Perry et al. [80].

### Anhydrobiosis experiment

All experiments were performed in 35 mm diameter plastic vented Petri dishes (experimental dishes) lined with white filter paper at the bottom. Each experimental dish was filled with 450 μl of distilled water and selected individuals (extracted from the environmental samples or culture) were transferred to the experimental dishes using an automatic pipette. Each dish contained seven specimens, and 10 dishes in total were established per species. The experimental dishes were then closed and placed in an environmental chamber with controlled conditions of 40–50% humidity and 20°C (PolLab, Q-Cell 140, https://www.pol-lab.eu/en/) and left to dry over the course of 72 hours. Later, the dishes were stored in the same chamber until the day of rehydration.

The drying process took 72 hours as it was necessary for tardigrades to form tuns of correct appearance, and that point was considered as the beginning of the experiment. Specimens were rehydrated after seven different timepoints of tun stage duration (0-, 7-, 14-, 30-, 60-, 120-, 240-days) each constituting a separate experimental group. In the time “0” group, specimens were rehydrated immediately after dehydration (i.e. 72 hours after transferring tardigrades to experimental dishes), whereas specimens from, for example, experimental group 7 were rehydrated after 7 days of tun stage duration. The procedure was repeated for each group.

**Fig 1:**
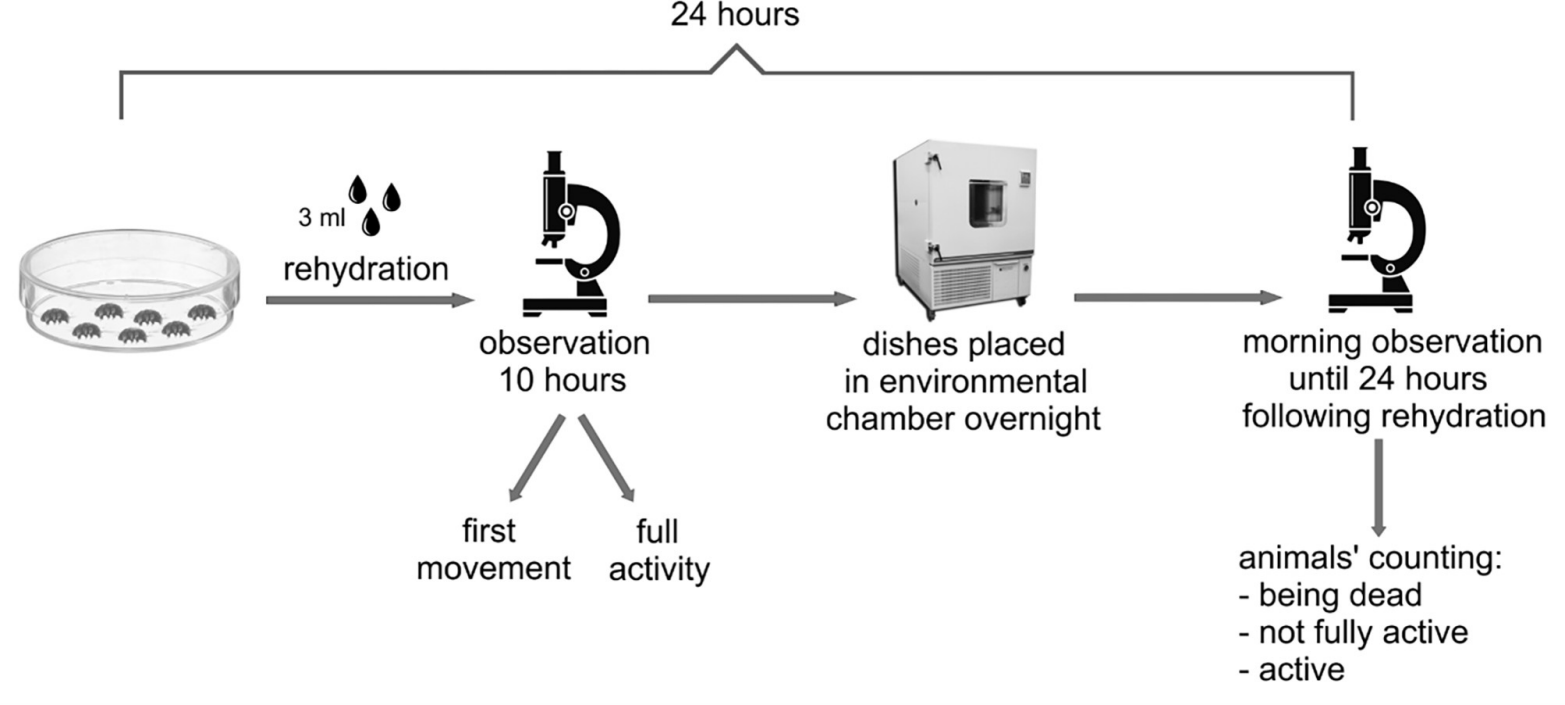
A simple schematic illustration of the experiment (according to Roszkowska et al.).

Rehydration was achieved by adding 3 ml of distilled water to each experimental Petri dish (Fig 1). Then, specimens were transferred using an automatic pipette to small glass cubes in which they were observed for 10 hours (at room temperature 24°C) under a stereomicroscope SZ51 or SZX7. After 10 hours, the cubes with non-moving and/or not fully active specimens were placed in an environmental chamber (40–50% humidity and 20°C) overnight. The next day, they were observed every 30 minutes until 24 hours following rehydration.

During the observation, the time of both the first movement and the return to full activity was noted. The first movement was defined as any visible sign of the movement of claws, legs, body, buccal tube, etc. Full activity was defined as coordinated movements of the body and legs, i.e. the start of crawling. Specimens observed to be fully active were removed from the cubes and no longer observed. All observations were completed after 24 hours and every not fully active specimen or those without any signs of movement (interpreted as being dead) were counted.

In total, seven experimental groups differing between the tun stage duration were prepared for each tardigrade species/population. Each experimental group consisted of 10 replicates of 7 specimens, which were scored at each time point. So, in total we used 70 specimens per experimental group, for each of the 7 experimental groups, for each tardigrade species/population, i.e. a total of 490 individuals of each species/population.

Seven parameters of survival and activity were measured and used to compare experimental groups and species/populations: (i) SA–success of anhydrobiosis (the number and percentage of undamaged specimens that formed correct tuns and survived anhydrobiosis) (ii) the number of non-moving (dead) tardigrades (NM); (iii) the number of individuals that did not reach full activity until the end of the observation (“not fully active”; NFA); (iv) the time required for the first movement of any first individual (FM); (v) the time required for the first movement of all individuals (FMA); (vi) the time required to return to full activity by the first specimen (FA), and (vii) the time required to return to full activity by all individuals (FAA) in a given experimental dish.

### Statistical analysis

**Fig 2:**
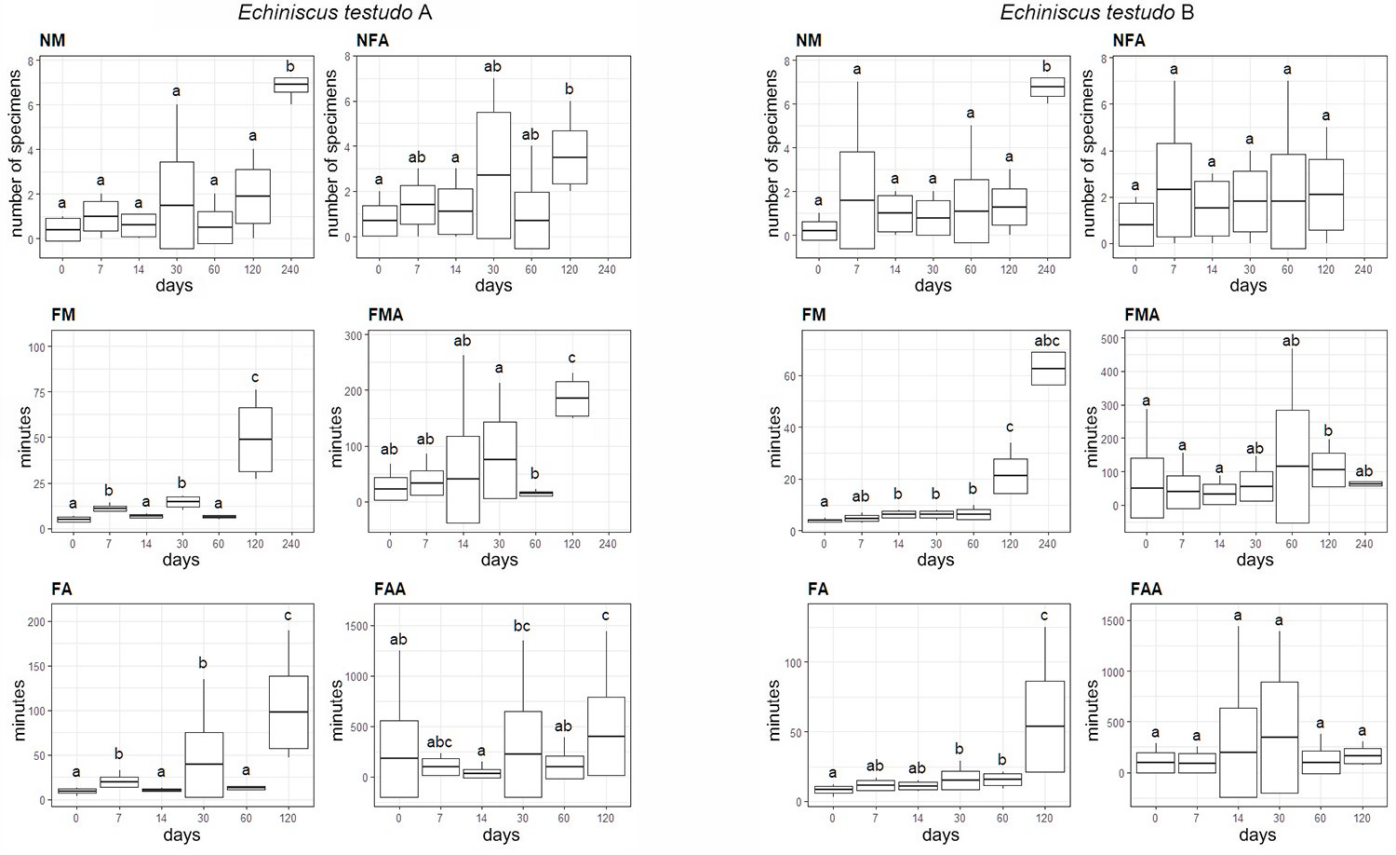
Differences in the number of nonmoving (NM) and not fully active (NFA) individuals, timing of the first movement of any individual (FM) and all moving individuals (FMA), full activity of any individual (FA) and full activity of all active individuals (FAA) between particular treatments (horiziontal axis—days in anhydrobiosis) in two populations of *Echiniscus testudo*: A—urban habitat, B—population from surroundings of the Wielkopolski National Park. Letters above the boxes (a, b, c, d) denote lack of significant (p < 0.05) differences between groups marked with the same letter.

**Fig 3:**
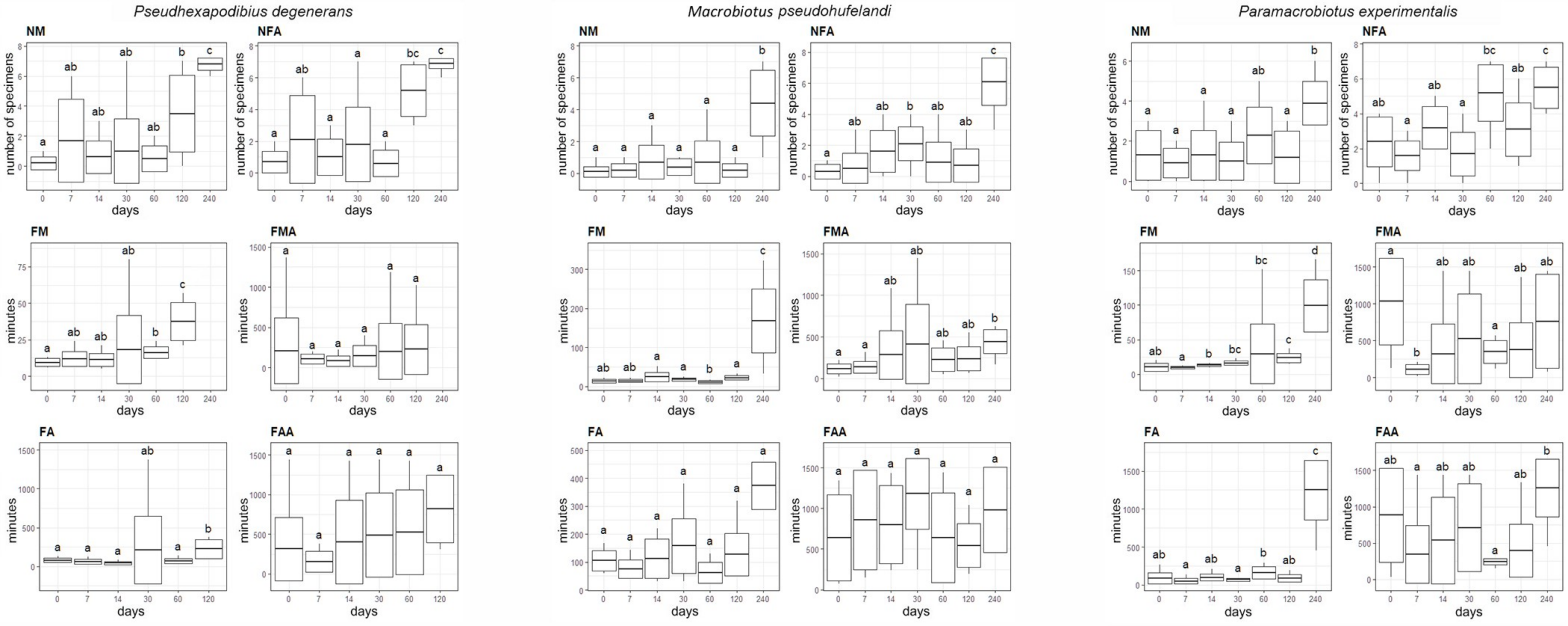
Differences in the number of nonmoving (NM) and not fully active (NFA) individuals, timing of the first movement of any individual (FM) and all moving individuals (FMA), full activity of any individual (FA) and full activity of all active individuals (FAA) between particular treatments (horiziontal axis—days in anhydrobiosis) in populations of three tardigrade species: *Pseudhexapodibius degenerans*, *Macrobiotus pseudokufelandi*, *Pararamacrobiotus experimentalis*. Letters above the boxes (a, b, c, d) denote lack of significant (p < 0.05) differences between groups marked with the same letter.

Each species/population was tested to check the influence of the time spent in anhydrobiosis (tun stage duration) on the values of each of the six survival and activity parameters (NM, NFA, FM, FMA, FA, FAA). Since the distribution of all recorded variables were far from normal, we used nonparametric tests to analyze the data. Comparisons between experimental groups was performed with the Permutational Analysis of Variance (Permanova, [81]) with 999 permutations. The Pairwise.adonis test for multiple comparisons adjusted with the Bonferroni correction was applied post hoc to compare differences between individual pairs of experimental groups (results were displayed in respective box-plots, Figs 2 and 3). The same test was later performed to compare differences between activity measures of the species/populations after additional stratification for experimental groups. To compare overall differences in activity measurements, a multivariate Permanova was also performed on a matrix consisting of all activity measures (overall test). Multivariate homogeneity of group dispersions was analyzed with the function betadisper to evaluate the dispersion of the measured values within each studied population [82]. When the results of the test were significant, the Permanova was performed with standardized, z-transformed data. All calculations were performed in R 4.0.2 [83] under RStudio 1.3.1056 with the use of ‘vegan’ package, while the graphs were produced with the ggplot2 package [84]. We considered *p* = 0.05 as the threshold determining statistical significance. Raw data used for all calculations are presented in S1 Appendix.

## Results

### General description of anhydrobiosis patterns

We chose the survival rate as the most basic measurement, because anhydrobiosis is induced to improve survival. Survival rates are given in Table 2. The highest survival rates (SA) were observed in groups with shorter tun stages, amounting to 0–60 days. The exceptions included *Mac*. *pseudohufelandi* and *Pam*. *experimentalis*, which exhibited quite high survival rate even after 120 days (97% and 86%, respectively). All the species demonstrated the lowest survival rates after 240 days of the tun (1–6% for *Ech*. *testudo*, 1% for *Psh*. *degenerans*), although *Mac*. *pseudohufelandi* and *Pam*. *experimentalis* featured higher survival rates of 240 days (38% and 43%, respectively).

**Table 2:** Values of applied measures of survival and activity used to estimate anhydrobiosis success.

***Echiniscus testudo (A)***							
**MA**	**NM**	**NFA**	**SA**	**FM (min.)**	**FMA (min.)**	**FA (min.)**	**FAA (min.)**
**TR**							
0	2	7	67 (100%)	3	287	3	287
7	16	23	54 (93%)	3	155	7	256
14	10	15	60 (88%)	5	87	7	1440
30	8	18	62 (90%)	4	145	9	1386
60	11	18	59 (87%)	5	467	9	380
120	13	21	57 (84%)	14	197	25	301
240	66	68	2 (6%)	58	67	0	0
***Echiniscus testudo* (B)**							
**MA**	**NM**	**NFA**	**SA**	**FM**	**FMA**	**FA**	**FAA**
**TR**							
0	4	7	65 (94%)	3	68	4	1244
7	10	14	60 (88%)	10	86	16	233
14	6	11	63 (93%)	6	262	9	149
30	15	27	55 (89%)	10	213	17	1350
60	5	7	65 (94%)	5	23	10	390
120	19	35	51 (76%)	27	231	47	1440
240	69	70	1 (1%)	96	96	0	0
***Pseudhexapodibius degenerans***							
**MA**	**NM**	**NFA**	**SA**	**FM**	**FMA**	**FA**	**FAA**
**TR**							
0	2	7	68 (99%)	6	1370	44	1440
7	17	21	51 (91%)	8	197	42	380
14	6	10	62 (95%)	5	222	15	1423
30	10	18	58 (97%)	7	402	42	1440
60	5	6	64 (93%)	12	1180	34	1422
120	35	52	32 (58%)	21	1027	101	1176
240	69	70	1 (1%)	63	63	0	0
***Macrobiotus pseudohufelandi***							
**MA**	**NM**	**NFA**	**SA**	**FM**	**FMA**	**FA**	**FAA**
**TR**							
0	1	3	66 (99%)	7	221	60	1343
7	2	5	67 (100%)	10	312	39	1451
14	7	16	61 (90%)	10	1083	30	1431
30	4	21	65 (94%)	12	1445	31	1565
60	7	9	62 (95%)	7	456	22	1440
120	2	7	68 (97%)	16	554	61	1043
240	43	60	26 (38%)	33	628	313	1352
***Paramacrobiotus experimentalis***							
**MA**	**NM**	**NFA**	**SA**	**FM**	**FMA**	**FA**	**FAA**
**TR**							
0	13	24	55 (100%)	5	1440	18	1413
7	9	16	60 (88%)	7	207	16	1440
14	13	32	53 (83%)	10	1440	62	1440
30	10	17	59 (87%)	13	1440	43	1440
60	23	52	46 (67%)	10	573	69	289
120	12	31	55 (86%)	15	1359	44	1332
240	39	55	29 (43%)	66	1440	452	1440

*TR*–treatment; *MA*–measures of activity; *SA*–success of anhydrobiosis (number of specimens and % of all found, not damaged and forming correct tun specimens); *NM*–number of non-moving (dead) tardigrades; *NFA*–number of individuals that didn’t reach full activity during time of observation; *FM*–time required for the first movement of any first individual; *FMA*–time required for first movement of all individuals; *FA*–time required for return to full activity of the first individual; *FAA*–time required for all individuals to return to full activity. Values given in table include all experimental replicate dishes within a given research group for a given duration of the tun stage.

The time needed to observe the first movement of any specimen (FM) was quite short in all species/populations in the case of shorter tun stage time points (0–60 days), longer after 120 days, and the longest after 240 days of the tun stage. The “fastest” species was *Ech*. *testudo* (A), which needed only approx. 3 to 5 min to show first movements. After 120 days spent in the tun stage, all species clearly needed more time. The fastest was *Ech*. *testudo* (A) which moved after 14 minutes, while the slowest *Ech*. *testudo* (B) needed 27 minutes for FM. The clearest differences in that parameter were visible after 240 days as the species needed between 33 minutes (*Mac*. *pseudohufelandi*) and 96 minutes (*Ech*. *testudo* (B)).

For some species the full activity of any specimen (FA) was observed after a very short time (*Ech*. *testudo*, 3–4 min. after the shortest tun stage). In contrast, others species required much more time to FA, even after short tun stage periods (0, 7 and 14 days). For example, *Mac*. *pseudohufelandi* needed 30 to 60 minutes (Table 2). Strikingly, after the longest tun stage, some species had no individuals that returned to full activity (*Ech*. *testudo* and *Psh*. *degenerans*), while others needed a long time to return (*Mac*. *pseudohufelandi*– 313 minutes, and *Pam*. *experimentalis–* 452 minutes).

We also measured the time of first movements and full activity of all specimens in each group, which allowed us to assess typical or standard behavior of the species. The time of the first movement and full activity of all specimens (FMA and FAA, respectively) was rather variable and ranged from 23 to 1,565 minutes, and thus, there was no visible pattern in relation to the time spent in anhydrobiosis (Table 2).

### Response to anhydrobiosis of two populations of heterotardigrade *Ech*. *testudo*

In our study, we compared anhydrobiotic abilities of specimens of *Ech*. *testudo* from two populations. Most of the measured parameters displayed only modest differences between the Wielkopolski National Park population A and Poznań urban population B. The number of non-moving (NM) individuals in both groups was very similar after 0–120 days of the tun stage, except the group that spent 240 days in the tun stage, where the NM numbers dropped significantly (Table 2). In the natural population A, only two individuals showed any signs of movement after 240 days spent in the tun stage, but they did not reach full activity. Similarly, in the urban population B, only one individual showed any signs of life after 240 days, and it also did not return to full activity (Table 2). Similar numbers of individuals from both populations failed to return to full activity (NFA), and no significant differences were observed between experimental groups except the 240- day experimental group (the significance of post-hoc tests consult markings in Fig 2). However, in the natural population A, the 120-day group had a significantly higher number of NFA specimens than experimental groups with shorter tun stages (Fig 2). The differences were not influenced by the differences in the dispersion (pairwise comparisons of group dispersions with Tukey HSD: p > 0.62).

The time before the first movement of any individual (FM) was very similar in both populations as there were no significant differences between experimental groups from 0–60 days. However, we recorded a clear FM difference in the 120-day group, and even a bigger one in the 240-day group. This significant difference was caused by a much higher dispersion of measurements in the 120-days treatment of both populations (Tukey HSD: p < 0.002 in all of the compared pairs), and it was no longer recorded when the test was carried out on standardized data (p = 0.141 and p = 0.661 for population A and B, respectively). Despite significant differences between experimental groups, especially in the urban population unaffected by dispersion (Tukey HSD: p > 0.57), the first movement of all individuals (FMA) was a far less informative parameter and did not show any clear trend among the studied populations (Fig 2).

Finally, the patterns of reaching full activity by the first individual and by all individuals (FA and FAA, respectively) were similar to what we observed for FM and FMA. In the 120-day experimental group, FA was clearly longer than in shorter experimental groups which exhibited only marginal differences between them, although this result was affected by dispersion differences in almost all cases p<0.049 and was no longer present after transformation, p > 0.8. None of the observed individuals reached full activity after 240 days in the tun stage (Fig 2). No clear trend in the timing of FAA was observed for either population.

### Response to anhydrobiosis of three eutardigrade species

General patterns in survival and activity measurements of the three eutardigrade species were very similar to patterns described for the two populations of *Ech*. *testudo*. Animals subjected to the longest tun stage (240 days) clearly differed from other experimental groups with respect to NM, NFA and FM (excepts the 240-day NFA did not differ from the 60-day of *Pam*. *experimentalis* and from the 120-day of *Psh*. *degenerans*). It did not result from differences in dispersion (p > 0.01), except *Mac*. *pseudohufelandi*, where measurements of FM after 240 days in the tun stage were significantly more dispersed (p < 0.0001), and they were not significant after standardisation (p = 0.089). In the case of *Pam*. *experimentalis* and *Mac*. *pseudohufelandi*, FA after 120 days in the tun stage did not differ significantly from other groups as it was observed among *Pse*. *degenerans* and both populations of *Ech*. *testudo*. For both species, the difference was observed between the 240-day group and the remaining ones (note that *Pse*. *degenerans* and *Ech*. *testudo* did not reach FA after 240 days). Again, no clear and universal pattern was observed in the case of FMA and FAA (Fig 3).

### Differences between studied populations

The values of tests that spotted differences between the five studied populations from the four tardigrade species were statistically significant for all of the measured parameters (in all analyses p < 0.001, except FMA with p = 0.011; values of FM, FMA, FA and FAA z-transformed due to the lack of variance homogeneity). These differences were the greatest in the case of NFA, with F_4,315_ = 16.829, R^2^ = 0.095 and the lowest, but still highly significant, in FMA: F_4,315_ = 2.365, R^2^ = 0.024.

Our models showed that the interaction between the species/population and the time spent in the tun stage was significant in the case of all the parameters (p < 0.001, F > 1.995, R^2^ > 0.119), with the exception of NM, which did not reach statistical significance (p = 0.062, F_24,315_ = 1.4198, R^2^ = 0.08538). With regard to the remaining parameters, our results indicate that the populations differ not only in their response to drying, but also the patterns of the response alter depending on the time spent in the tun stage.

The two studied populations of *Ech*. *testudo* did not differ significantly in their overall anhydrobiosis capability (multivariate model: p = 0.21, F_1,116_ = 1.066, R^2^ = 0.009). However, significant differences between these populations were observed when comparing FM (p < 0.001, F_1,121_ = 7.340, R^2^ = 0.058) and FA (p < 0.001, F_1,116_ = 3.037, R^2^ = 0.026). The remaining pairs of tardigrade populations differed significantly in their overall anhydrobiosis capability (p < 0.001, F > 5.574, R^2^ > 0.043) (Table 3). The differences were most prominent between *Ech*. *testudo* (A) vs *Psh*. *degenerans*, and *Ech*. *testudo* (A) vs *Mac*. *pseudohufelandi*, because they varied strongly in all but one measured parameters (NFA and FM, respectively). The smallest number of significant differences was observed between *Psh*. *degenerans* and *Pam*. *experimentalis*, which were recorded when comparing NM, NFA and FMA parameters (see Table 3).

**Table 3:** Differences in the applied activity measures and the measures combined between pairs of populations.

**NM**					
**Species**	*Ech*. *testudo* (A)	*Psh*. *degenerans*	*Mac*. *pseudohufelandi*	*Ech*. *testudo* (B)	*Pam*. *experimentalis*
*Ech*. *testudo* (A)	-	0.002	0.001	0.945	0.168
*Psh*. *degenerans*	6.554	-	0.078	0.008	0.001
*Mac*. *pseudohufelandi*	11.829	2.334	-	0.001	0.001
*Ech*. *testudo* (B)	0.040	5.607	10.685	-	0.174
*Pam*. *experimentalis*	1.455	12.633	21.152	1.666	-
**NFA**					
**Species**	*Ech*. *testudo* (A)	*Psh*. *degenerans*	*Mac*. *pseudohufelandi*	*Ech*. *testudo* (B)	*Pam*. *experimentalis*
*Ech*. *testudo* (A)	-	0.41	0.002	0.417	0.002
*Psh*. *degenerans*	3.069	-	0.15	0.252	0.001
*Mac*. *pseudohufelandi*	6.626	1.37	-	0.033	0.001
*Ech*. *testudo* (B)	0.588	0.986	3.359	-	0.001
*Pam*. *experimentalis*	5.868	13.697	23.223	8.825	-
**FM**					
**Species**	*Ech*. *testudo* (A)	*Psh*. *degenerans*	*Mac*. *pseudohufelandi*	*Ech*. *testudo* (B)	*Pam*. *experimentalis*
*Ech*. *testudo* (A)	-	0.001	*0*.*15*	0.001	0.001
*Psh*. *degenerans*	27.506	-	0.087	0.001	0.639
*Mac*. *pseudohufelandi*	*1*.*6662*	3.859	-	0.001	0.215
*Ecg*. *testudo* (B)	7.340	4.939	13.617	-	0.001
*Pam*. *experimentalis*	35.406	1.731	0.596	8.91	-
**FMA**					
**Species**	*Ech*. *testudo* (A)	*Psh*. *degenerans*	*Mac*. *pseudohufelandi*	*Ech*. *testudo* (B)	*Pam*. *experimentalis*
*Ech*. *testudo* (A)	-	0.001	0.001	0.375	*0*.*179*
*Psh*. *degenerans*	17.547	-	0.001	0.001	*0*.*012*
*Mac*. *pseudohufelandi*	51.832	18.392	-	0.001	*0*.*14*
*Ech*. *testudo* (B)	0.665	23.92	58.313	-	*0*.*936*
*Pam*. *experimentalis*	*1*.*6204*	*4*.*2093*	*2*.*0324*	*0*.*1254*	-
**FA**					
**Species**	*Ech*. *testudo* (A)	*Psh*. *degenerans*	*Mac*. *pseudohufelandi*	*Ech*. *testudo* (B)	*Pam*. *experimentalis*
*Ech*. *testudo* (A)	-	0.001	0.001	0.001	*0*.*162*
*Psh*. *degenerans*	103.43	-	0.007	0.001	0.512
*Mac*. *pseudohufelandi*	135.29	6.022	-	0.001	0.164
*Ech*. *testudo* (B)	3.037	56.388	79.949	-	*0*.*092*
*Pam*. *experimentalis*	*1*.*7118*	51.363	2.067	*2*.*4231*	-
**FAA**					
**Species**	*Ech*. *testudo* (A)	*Psh*. *degenerans*	*Mac*. *pseudohufelandi*	*Ech*. *testudo* (B)	*Pam*. *experimentalis*
*Ech*. *testudo* (A)	-	0.001	*0*.*001*	0.244	*0*.*002*
*Psh*. *degenerans*	21.247	-	0.001	0.001	0.15
*Mac*. *pseudohufelandi*	*11*.*582*	18.905	-	*0*.*001*	0.012
*Ech*. *testudo* (B)	1.0939	21.499	*9*.*6818*	-	*0*.*01*
*Pam*. *experimentalis*	*7*.*5874*	2.960	6.447	*5*.*4377*	-
**All parameters combined (overall test)**					
**Species**	*Ech*. *testudo* (A)	*Psh*. *degenerans*	*Mac*. *pseudohufelandi*	*Ech*. *testudo* (B)	*Pam*. *experimentalis*
*Ech*. *testudo* (A)	-	0.001	*0*.*001*	0.21	*0*.*001*
*Psh*. *degenerans*	23.859	-	0.001	0.001	*0*.*001*
*Mac*. *pseudohufelandi*	*11*.*419*	16.144	-	*0*.*001*	0.002
*Ech*. *testudo* (B)	1.066	22.448	*10*.*048*	-	0.001
*Pam*. *experimentalis*	*9*.*7417*	*6*.*4766*	5.574	42.257	-

Upper diagonal: *p* values; Lower diagonal: F values; *NM*–number of non-moving (dead) tardigrades; *NFA*–number of individuals that didn’t reach full activity during time of observation; *FM*–time required for the first movement of any first individual; *FMA*–first movement of all individuals; *FA*–time required for return to full activity of the first individual; *FAA*–time required for return to full activity of all individuals; italics–data standardised before the analysis.

## Discussion

Our results deal with the length of the anhydrobiotic state in which tardigrades were able to survive. This duration helped us indicate tardigrade anhydrobiosis patterns. The experiments consisted in a long-term anhydrobiosis of four tardigrade species in controlled laboratory conditions. We analyzed their ability to recover from the tun stage on the basis of survival and activity measures of anhydrobiosis success.

First, we observed differences in the survival rate between the tested species. The highest survival rate (more than 80% and without any visible decrease) was observed among *Ech*. *testudo* population B (urban) and *Psh*. *degenerans*, because they survived for 0–60 days in the tun stage, as well as *Ech*. *testudo* population A (natural), *Mac*. *pseudohufelandi* and *Pam*. *experimentalis*, which spent 0–120 days in the tun stage. This survival is correlated with a very low number of non-moving/dead (NM) specimens from experimental groups that survived from 0 to 120 days. The lack of a decrease in the survival rate with the increased duration of the tun stage was also reported for *Pam*. *richtersi* [66]. However, in that experiment naturally desiccated tardigrades were rehydrated after a period of under 21 days. Even more spectacular results were reported for *Mil*. *inceptum* and *Ram*. *subanomalus* which exhibited no significant change in the survival rate even after 240 days in the tun stage [65]. Strikingly, we observed a drastic decrease in their survival after 240 days in the tun stage for all of the studied species. More specifically, only a few percent of specimens of *Ech*. *testudo* (only 3% in total in both populations) and *Psh*. *degenerans* (1%) and about 40% of *Mac*. *pseudohufelandi* and *Pam*. *experimentalis* survived after 240 days spent in the tun stage.

Interspecific variation of tardigrade survival in the tun stage is well known (e.g. [53]). The proposed reasons include body size and/or energetic conditions. However, the controversial discussion about body size and anhydrobiotic survival of tardigrades [52, 58] is also negated by contrasting survival within Macrobiotidae with similar body sizes, because *Pam*. *experimentalis* and *Mac*. *pseudohufelandi* show higher survival rates, whereas *Psh*. *degenerans* survival is low after 240 days in the tun stage. A very high survival of 240 days in the tun stage was also reported for *Mil*. *inceptum* and *Ram*. *subanomalus*, which are described as medium sized/larger species [65]. A possible explanation for the variation in anhydrobiosis survival may be explained by the energy required to enter, sustain, and leave the tun stage [58]. However, this hypothesis does not correspond to the experiments presented in the current study, even though body size and energetic condition are assumed to affect the probability of anhydrobiosis survival [58].

A low survival rate observed for *Ech*. *testudo* in the current study was surprising when compared to other tested species in our experiment, given that specimens of that species had been previously observed to return to activity after about 3 or even about 20 years in the tun stage [22, 74]. However, it should be remembered that Rebecchi et al. [22] and Jørgensen et al. [74] applied semi-natural drying in moss samples, whereas we used artificial drying on filter paper, which certainly mattered when the species were recovering from anhydrobiosis. Therefore, our research results should not be compared with those by Rebecchi et al. [22] and Jørgensen et al. [74]. Apart from different dehydration protocols, the variations may have been caused by phenotypic plasticity of the species that responded to microclimatic conditions. The results provided by Faurby et al. [53] suggesting that populations of the same species from different localities may differ in anhydrobiotic capabilities should also be taken into consideration.

The time needed for the first movement of any specimen (FM) from all of the studied species, especially in the short tun stage, was very short (maximum a few minutes). However, it was much longer after 120 days and especially long after 240 days in the tun stage. The fastest species was *Ech*. *testudo* specimens which needed only approx. 3 to 10 minutes, while other species needed approx. 6 to 13 minutes. The return to full activity (FA) of the first specimen was also very fast (after the shortest duration of anhydrobiosis) for some species, e.g. *Ech*. *testudo* 3–4 minutes, while other species needed much more time to perform FA, e.g. it took *Mac*. *pseudohufelandi* even several dozen minutes. After the longest time spent in the tun stage (240 days), none of the specimens of *Ech*. *testudo* and *Psh*. *degenerans* returned to full activity during a 24-hour observation following dehydration. For other species the time of the return was very long 5–7 hours (*Mac*. *pseudohufelandi* and *Pam*. *experimentalis*). From the ecological point of view, it is more beneficial for species to return to activity faster, making use of the short time of water availability and the earlier start of feeding or/and reproduction. Besides, species that require more time to return to full activity have more time to repair possible cellular and/or molecular damages (see below). With regard to the return to full activity after 240 days of anhydrobiosis, we need to emphasize that the artificial drying protocol with specimens kept in anhydrobiosis in artificial conditions (filter paper) does not fully replicate natural conditions of the species or environmental conditions of the habitats from which they were collected. The specimens we collected, which had never been exposed to 240-day droughts in their natural environment, may have simply failed to cope with unusual stress.

The differences between the five populations of the four tardigrade species were statistically significant for all measures of activity. The interaction between the species and the time spent in the tun stage indicates that the populations differ not only between their response to drying, but also patterns of this response vary depending on the time spent in the tun stage. Another interesting observation is that the two populations of *Ech*. *testudo* do not differ significantly when their overall reaction to anhydrobiosis is compared, however, they do differ in their timing of FM and FA. Again, it corresponds to the results of Faurby et al. [53], which means that populations of the same species from different localities may differ in anhydrobiotic capabilities. Nevertheless, the specific background of these differences remains unknown.

It is clearly visible that the longer the tun stage, the more time a species needs to return to full activity. However, according to the present study and the previous publication by Roszkowska et al. [65], it can be concluded that this relation is far from linear. This observation is in agreement with an explanation that the time spent in the tun stage is correlated with the number of damages which have to be repaired, as well as, the time required to restore metabolism [see e.g. 23, 49, 51]. Accordingly, the time necessary to reach full activity after a shorter tun stage is relatively short for all species/populations (within the range of minutes). Such a rapid response in nature may represent an adaptation to brief periods of liquid water appearing in tardigrades’ natural habitats. Moss cushions, lichens, and soil itself (at least on the surface) may absorb water in the early morning when fog and dew appear. When mosses, lichens and topsoil subsequently dry out as the sun rises, there is a rapid decline of humidity in both the air and the substrate. Thus, the liquid water phase in these habitats can last only for a few hours per day or even shorter, with longer hydroperiods only occurring occasionally during rainy weather. However, even after the rain, especially in moderate or dry climatic zones, water is frequently available only for a few hours or a few days. Thus, rapid recovery from anhydrobiosis can be regarded as an adaptation to temporary habitats, since in such short time tardigrades must supplement energy resources exploited during anhydrobiosis (e.g. [85]) and carry out their entire life cycle. This could also suggest that the ability to return to active life faster than other tardigrades is a favorable trait, because fast-recovering animals can begin feeding and reproducing earlier, exploiting potentially limited local resources and ensuring greater reproductive success.

Finally, our study also shows that a predatory species (*Pam*. *experimentalis*) needs more time to return to activity after the tun stage than a herbivorous species (*Ech*. *testudo*), an observation which contradicts Roszkowska’s et al. [65] findings explained by a prey-predator strategy. However, contrary to the research by Roszkowska et al. [65], we did not collect herbivorous and predator species from the same samples, and the species belonged to different classes (Eutardigrada and Heterotardigrada). In this case, the taxonomic status may play the main role, especially due to different anhydrobiotic machinery in Hetero- and Eutardigrada [86, 87]. Nonetheless, we agree that this hypothesis is interesting and should be considered, but given that we compare two different Tardigrada classes, more research comparing tardigrade species within the same class should be conducted so as to support this hypothesis.

In conclusion, we showed clear differences between anhydrobiotic abilities of different species/populations, which are reflected by the observed patterns. The patterns appear to be determined mainly by the time spent in the tun stage, but other factors, such as diet or interspecific variation (phenotypic plasticity triggered by habitat of origin) may also play a role. Following previous reports, we found that the critical factor is the duration of the tun stage, inversely correlated with successful anhydrobiosis and the time required for recovery from anhydrobiosis. The underlying mechanisms of this tendency are still not fully understood, but they are likely to be related directly to the taxonomic status and ecological strategies supported by metabolism.

## Supporting information

S1 Appendix The raw data used for all statistical calculations. (XLSX)
