# Correction: How long can tardigrades survive in the anhydrobiotic state? A search for tardigrade anhydrobiosis patterns

**DOI:** 10.1371/journal.pone.0317780

**Published:** 2025-01-15

**Authors:** The PLOS ONE Staff

The fourth author’s name is spelled incorrectly. The correct name is: Zofia Książkiewicz. The correct citation is: Roszkowska M, Gołdyn B, Wojciechowska D, Książkiewicz Z, Fiałkowska E, Pluskota M, et al. (2023) How long can tardigrades survive in the anhydrobiotic state? A search for tardigrade anhydrobiosis patterns. PLoS ONE 18(1): e0270386. https://doi.org/10.1371/journal.pone.0270386.

There are errors in the author affiliations. The correct affiliations are as follows:

Milena Roszkowska^1,2^, Bartłomiej Gołdyn^3^, Daria Wojciechowska^4^, Zofia Książkiewicz^3^, Edyta Fiałkowska^5^, Mateusz Pluskota^3^, Hanna Kmita^2^, Łukasz Kaczmarek^1^

**1** Department of Animal Taxonomy and Ecology, Faculty of Biology, Adam Mickiewicz University, Poznań, Poland, **2** Department of Bioenergetics, Institute of Molecular Biology and Biotechnology, Faculty of Biology, Adam Mickiewicz University, Poznań, Poland, **3** Department of General Zoology, Faculty of Biology, Adam Mickiewicz University, Poznań, Poland, **4** Department of Macromolecular Physics, Faculty of Physics, Adam Mickiewicz University, Poznań, Poland, **5** Institute of Environmental Sciences, Jagiellonian University, Kraków, Poland.

The publisher apologizes for the errors.
